# Cigarette Smoking

**Published:** 1883-07

**Authors:** 


### CIGARETTE SMOKING.

SIR HENRY THOMPSON writes to the London *Lancet:* I think I can offer you a practical hint of some value in connection with cigarette smoking.

First, the cigarette, without a mouthpiece, is really never smoked more than half way through in the East, where cigarettes are very cheap. It is well understood there, as it is by all practiced cigarette smokers, that every inhalation from a cigarette slightly deteriorates in quality from the first. A small deposit of the very offensive oil of tobacco is deposited in the finely cut leaf, which acts as a strainer, and intercepts the deposit as it passes. Very little of this arrives in the smoker's mouth if he stops when half is consumed. I have seen many Oriental smokers who consume no more than a third. Turkish ladies, for example, as I have had personal opportunities of observing at Constantinople, will smoke fifty or upward in a day, but I need scarcely say, only in the manner I have described.

Secondly, if a cigarette with a card mouthpiece is employed, the noxious matter may be intercepted by always introducing a light plug of cotton wool into the tube. If now the cigarette is nearly consumed, a considerable quantity of brown and very offensive matter will be found in the cotton wool, from the evil of which the smoker is thus preserved.

Thirdly, some years ago I designed a cigarette holder or mouthpiece which opened transversely in the middle, disclosing a small cavity, which is filled with cotton wool. It would surprise many people, perhaps, to find that the result of smoking six cigarettes only in this tube is that this plug is saturated with a brown fluid like treacle, of powerfully offensive odor, and disagreeable almost beyond belief. The wool then requires to be changed, and in this manner the evil of smoking is very greatly diminished. Several of these were constructed by a well-known tobacconist close to your office in the Strand.

Lastly, the maximum of pernicious influence which occurs through cigarette smoking is attained by the practice of inhaling the smoke largely direct into the lungs, where it comes into immediate contact with the circulation, and the toxic effect is so strongly perceptible after three or four consecutive inhalations, and so felt by a sensitive person to the very tips of the fingers. I have no doubt that the effect would in most cases be notably recorded by the sphygmograph. Such smoking is, of course, or ought to be, exceptional. All the fragrance, with a little only of the toxic effect, is obtained by admission of the smoke into the mouth only, still more by passing it through the passages of the pharynx and nose; but it is the pulmonary inhalation referred to, often associated with cigarette smoking, and rarely with the pipe, which constitutes the chief mischief of the cigarette. I may say, in passing, that the well-known Oriental method of smoking, practiced by means of the narghileh, in which the smoke, though drawn through water, passes invariably through the lungs, explains the powerful effects which sometimes unpleasantly surprise novices in its use.

Smoked, then, simply, and with cotton wool interposed, I do not hesitate to regard the cigarette as the least potent, and therefore the least injurious, form of tobacco smoking. Without this precaution it may be made, although not necessarily, the most ready means of conveying the active principle of tobacco, by means of smoke, into the system.

